# Pain as clinical manifestations of COVID-19 infection and its management in the pandemic era: a literature review

**DOI:** 10.1186/s41983-020-00258-0

**Published:** 2020-12-27

**Authors:** I. Putu Eka Widyadharma, Ni Nyoman Shinta Prasista Sari, Kadek Enny Pradnyaswari, Kadek Tresna Yuwana, I. Putu Gede Danika Adikarya, Clarissa Tertia, I. A. Sri Wijayanti, I. A. Sri Indrayani, Desak Ketut Indrasari Utami

**Affiliations:** 1 Department of Neurology, Faculty of Medicine, Udayana University/Sanglah Hospital, Bali, Indonesia; 2 Medical Student, Faculty of Medicine, Udayana University/Sanglah Hospital, Bali, Indonesia

**Keywords:** COVID-19, Muscular pain, Neuropathic pain, Management

## Abstract

Coronavirus disease 2019 (COVID-19) is a current global pandemic. The case number has increased since December 31, 2019. It has been reported that COVID-19 patients have been giving pain complaints, one of which is muscular pain. Other types of pain that have also been reported by COVID-19 patients are joint pain, stomach pain, and testicular pain. Neuropathic pain is the rarest case among others. COVID-19 mechanisms in the nerve and musculoskeletal damage are believed to be caused by the expression and distribution of angiotensin-converting enzyme 2 (ACE-2). Patients with pain, especially neuropathic pain, normally do not respond well to various therapies, and experience psychiatric disorders such as depression, which leads to a decrease in the patient’s quality of life. Important considerations for health professionals in terms of pain management during this pandemic include ensuring treatment continuity, painkillers, utilization of telemedicine, biopsychosocial management approach, and modifying therapy needs to reduce the risks of COVID-19 complications.

## Introduction

Coronavirus disease 2019 (COVID-19), which originally came from Wuhan, China, is currently considered as a significant threat towards global health. Cases have increased since December 31, 2019, until January 3, 2020, as indicated from the total of 44 reported cases in China. Within the first 2 months of the pandemic, this virus has rapidly spread across countries and across the globe. On March 8, 2020, a total of 80,868 confirmed cases and 3101 deaths was reported by the National Health Commission of China, and the other 90 countries were also affected [1]. The World Health Organization (WHO) confirmed COVID-19 as a global pandemic on March 11, 2020. According to the data from WHO on May 13, 2020, in Indonesia, a total of 14,749 COVID-19 cases and 1007 deaths has been confirmed. Accordingly, the total number of worldwide cases reached 4,170,424, and 287,399 deaths were also confirmed. In February 2020, WHO announced that coronavirus disease (COVID-19) is caused by severe acute respiratory syndrome coronavirus [2]. This virus can be transmitted from person to person, so the spread becomes more aggressive. COVID-19 transmission occurs through droplets produced by coughing and sneezing [3]. The incubation period of COVID-19 ranges from 1 to 14 days [4]. Dry cough, fever, and fatigue are the most common symptoms for mild and intermediate patients. Besides that, patients also complain of having myalgia, abdominal pain, sore throat, headache, nasal congestion, and arthralgia [5–7].

Pain is an unpleasant sensory and emotional experience which is related to potential tissue damage [8]. Clinical manifestations of COVID-19 pain vary from headache, abdominal pain, arthralgia, to myalgia. Muscle pain or myalgia is one of the most frequent symptoms among COVID-19 patients, while neuropathic pain is rarely reported by COVID-19 patients. Patients with pain, particularly neuropathic pain normally do not respond well to various therapies. Moreover, they often experience psychiatric disturbances at the same time, such as depression, which leads to reduced quality of life of the patients [9, 10]. The purpose of this study is to discuss about pain which is commonly present in COVID-19 patients and its management.

### Musculoskeletal pain among COVID-19 patients

Muscle pain (myalgia) is one of the most frequent symptoms among COVID-19 patients besides having fever, cough, and dyspnea [11]. A meta-analysis study which involved 59,254 COVID-19 patients from 11 countries revealed that muscle pain occurred to 36% of the patients [11]. Another meta-analysis study in China mentioned that the prevalence of muscle pain was 21.9% from 8697 patients [12]. Nevertheless, two other studies located in Wuhan showed that the prevalence of muscle pain was 34.8% (48 out of 138 patients) and 11% (11 out of 99 patients) [5, 13]. In Europe, the clinical manifestation of muscle pain was found on a higher percentage, in a study of 1420 patients, 62.5% of them experienced muscle pain [14]. Meanwhile, in South Korea, the clinical manifestation of muscle pain was only 14.3% [15].

Joint pain (arthralgia) can occur in COVID-19 patients with a prevalence of 10–15% [16]. Joint pain (arthralgia) was also mentioned as one of the possible early symptoms among COVID-19 patients [17]. In a study conducted by Cipollaro and colleagues, it was found that the prevalence of arthralgia and/or myalgia was 15.5% with total data of 12,046 patients [18]. A study in China revealed that 14.9% of the patients experienced either muscle pain or joint pain (164 out of 1099 patients) [9].

Clinical manifestations of muscle pain caused by COVID-19 were more likely to be experienced by adult patients rather than pediatric patients. A study categorized muscle pain or myalgia as a skeletal muscle injury. Skeletal muscle injury is defined as a condition where the patients experience musculoskeletal pain and an increase in serum creatinine kinase levels greater than 200 U/L [9]. In a study involving 214 patients of COVID-19, 23 patients (10.7%) experienced skeletal muscle injury. In severe cases, the prevalence of skeletal muscle injury was found to be higher, namely 17 patients (19.3%), while in non-severe cases, only 6 patients (4.8%) were reported [19]. Similar results were also found in another study where the prevalence of muscle pain was more likely to be found in severe cases, namely 17.3%, while in non-severe cases, the percentage was 14.5% [9].

Patients with skeletal muscle injury are also said to have higher creatinine kinase levels, higher neutrophil counts, lower lymphocyte counts, higher levels of C-reactive protein, and higher levels of D-dimers [19]. In addition, patients with skeletal muscle injury were found to have multi-organ injuries, including serious liver problems and kidney problems [19].

The incidence of musculoskeletal pain is associated with increased inflammatory responses. An increase in proinflammatory cytokines may induce the formation of prostaglandin E2, which is a pain mediator with its effect on peripheral pain receptors [20]. A significant increase in pro-inflammatory cytokines can cause cytokine storms. Cytokine storm is an uncontrolled systemic inflammatory response resulting from the release of large amounts of pro-inflammatory cytokines and chemokines by immune effector cells in SARS-CoV-2.21 infection [21].

In a study, severe cases were found to have higher levels of interleukin 2R (IL-2R), IL-6, IL-10, and tumor necrosis factor α (TNF-α) rather than the moderate cases [22]. These results are supported by another study which pointed out that those levels of pro-inflammatory cytokines were also found to be higher in severe cases [23]. High expression of pro-inflammatory cytokines and chemokines, especially in severe cases, results in a worsening inflammatory response and leads to tissue damage and organ failure. Thus, this cytokine storm is associated with disease severity [22, 23].

Another mechanism regarding the occurrence of musculoskeletal pain is likely to be related to ACE-2 that is determined as SARS-CoV2’s functional receptor, which can be found in the skeletal muscle [24]. ACE-2 is also a functional receptor for SARS-CoV. However, in the postmortem examination of SARS-CoV, ACE-2 was not detected in the skeletal muscle [25]. Hence, it is necessary to learn more about how SARS-CoV-2-binding ACE2 can infect the muscle and joint cells.

### Abdominal pain and testicular pain among COVID-19 patients

Acute abdominal pain is very common in the complaints of patients in the hospital. Acute abdominal pain can also be one of the symptoms for sufferers with COVID-19 [26]. ACE2 receptors are also found in the small intestine, which in studies often found symptoms of diarrhea in sufferers of COVID 19. In addition, in the stool examination, SARS-CoV RNA was found. Chest CT scan found ground-glass opacity, consolidation, and diagnosed with COVID-19 [27]. One reported case in the USA revealed that there was a COVID-19 patient who had atypical symptoms, namely abdominal pain, testicular pain, and back pain. Pain is described as a persistent stabbing pain that originates in the groin and migrates to the abdomen, pelvis, back, and chest. Two days before these symptoms appeared, the patient had a subjective fever. However, the patient denies any complaints of rhinorrhea, sore throat, coughing, shortness of breath, nausea, or vomiting. CT scans of the abdomen and pelvis reveal pulmonary ground-glass opacification, pneumonia type of consolidation, potential sigmoid colitis, and distal descending colon [28].

In a case report of a 43-year-old male patient with a history of type 1 diabetes mellitus infected with COVID-19, complained of testicular pain especially on the left side with swelling of the inguinal lymph nodes, but no scrotum redness was seen. On CT scan, regular enhancements were found on the testes, epididymis, testicular artery, and pampiniformis plexus. An ultrasound found swelling of the epididymis which supported the diagnosis of epididymitis. In a study conducted by Xu and colleagues in 2006, it was said that the coronavirus family (SARS-CoV) can cause damage to the testes and germ cells. The study of Cardona Maya, Fan, Liu, Wang, and colleagues, found high expression of ACE2 in the testicular tissue which can damage the testes and cause orchitis [29].

The appearance of abdominal pain symptoms is associated with two-way communication between the gut microbiota and the brain, namely between the endocrine (cortisol), the immune system (cytokines), and nerves (vagus nerve and enteric nervous system) [30]. In the case of COVID-19, the immune system has the biggest role in causing pain. ACE2, which is the target of the COVID-19 virus, can also be found in the human intestine. One study showed that ACE2 expression was mainly located on the luminal surface of the redifferentiated small intestinal epithelial cells, whereas lower expression was observed in crypt and colon cells [31]. They also linked the transport function of the amino acid ACE2 to the microbial ecology of the digestive tract, where the ACE2 mutant showed decreased expression of antimicrobial peptides and changes in microbial composition [31].

### Neuropathic pain among COVID-19 patients

Neuropathic pain is characterized by pain that arises from a lesion or disease that affects the somato-sensory system [10]. Neuropathic pain can result from an underlying disease, such as peripheral neuropathy, spinal cord disorders, multiple sclerosis, stroke, and even infection. These underlying illnesses often cannot be treated, and the neuropathic pain that accompanies them also requires long-term treatment. Neuropathic pain patients usually do not respond well to various therapies and are often accompanied by psychiatric disorders, such as depression, which leads to a decrease in the patient’s quality of life [32].

Allodynia, hyperalgesia, and spontaneous pain are typical symptoms of neuropathic pain. Allodynia appears to involve thick afferent fibers (Aβ), while damage to the peripheral sensory neurons makes the hyperalgesia and spontaneous pain. Although pain is one of the body protection, the nociception has to be controlled properly [32]. A study conducted at 13 hospitals in Indonesia reported five main clinical symptoms of neuropathic pain sufferers, namely, a prickling sensation (*n* = 589; 33.1%), a sensation such as electric shock (*n* = 542, 30.5%), burns (*n* = 407, 22.9%), paresthesia (*n* = 401, 22.5%), and hyperalgesia (*n* = 351, 19.7%) [33].

There have not been many reports regarding the symptoms of neuropathic pain among COVID-19 patients. A retrospective observational study on 214 COVID-19 patients at a hospital in Wuhan, China, examined the neurological manifestations in COVID-19 patients. Neurological manifestations are classified into the central nervous system such as headache, decrease of consciousness, acute cerebrovascular disease, and convulsions and peripheral nervous system such as taste disturbances, olfactory disorders, visual disorders, nerve pain, and manifestations of skeletal muscle injury. The subjects were divided into 2 groups, namely severe and moderate degrees of COVID-19, according to the American Thoracic Society guidelines for community-acquired pneumonia (CAP). This study reported that complaints of nerve pain among COVID-19 patients were found only in 5 out of 214 (2.3%) patients, specifically 4 patients were in the severe symptom group, and 1 patient was in the mild symptom group. However, in this study, no further definition of nerve pain was used [20].

ACE2 was identified as a functional receptor for COVID-19, including the nervous system and skeletal muscles which causes neurological manifestation [25, 35]. This has been confirmed in other CoV infections such as SARS-CoV and MERS-CoV. Studies found the SARS CoV nucleic acid in the patients’ cerebrospinal fluid and in the brain tissue. By encoding the mRNA of several COVID-19 virus proteins, such as SARS-CoV, it uses the S1 protein spike which allows the binding of virions to the cell membrane by interacting with the host ACE2 receptor [35, 36].

**Fig. 1:**
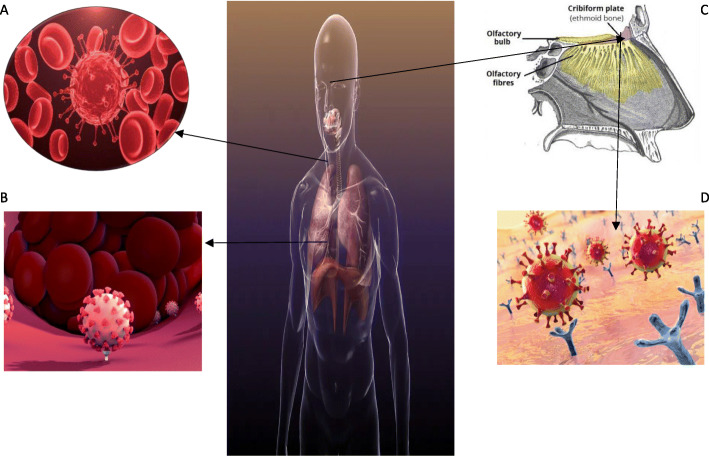
The mechanism of COVID-19 attacking the nervous system through ACE2 distribution [34]. (**a**) COVID-19 virus enter the brain circulation. (**b**) The virus particles will damage the endothelial lining thus supporting the virus to go through the brain. (**c**) COVID-19 virus will spread to the circulation or via the cribriform plate of the ethmoid bone. (**d**) The virus then will interact with the ACE2 receptor

COVID-19 spread to the circulation or via the cribriform plate of the ethmoid bone. This circulating COVID-19 virus then enters the brain circulation which facilitates the interaction of the COVID-19 virus spike protein with ACE2 that expressed in capillary endothelium. Furthermore, the virus particles will damage the endothelial lining, thus supporting the virus to go through the brain. Once the viral come to the neuron, they will interact with the ACE2 receptor which expressed in the neurons that can stimulate the viral growth accompanied by neuronal damage. These processes are all shown in Fig. 1. COVID-19 virus can reach and infect the brain through a cribriform plate close to the olfactory bulb. This statement is supported by several case reports with symptoms of changes in the sense of smell or hyposmia among COVID-19 patients [35, 37]. In subsequent studies, it was shown that ACE2 binds to the ectodomain of COVID-19 spike protein 10–20 times higher than spike protein in SARS-CoV [36].

There are other mechanisms reported regarding coronavirus infection leading to nervous system damage, which are listed as follows:

1. Direct infection injury

The protein and genetic material of the virus can be detected in cerebrospinal fluid or brain tissue, indicating the damaged nervous system caused by viruses [38].

2. Blood circulation pathways

An example of an atypical virus that enters the CNS through the blood circulation is the Japanese Encephalitis virus, through mosquito bites. Then, it is released into the blood to replicate in mononuclear macrophages throughout the body which then increases the blood-brain barrier’s permeability, thereby prompting entrance of the virus into the brain and cause viral encephalitis [39]. Studies have conducted that SARS CoV-2 rarely attacks the nervous system through this pathway [40].

3. Neuron pathways

Viruses infect sensory or motor nerve endings retrogradely or anterogradely via the motor proteins namely dynein and kinesin [41]. Olfactory neuron transport is one of the neuronal pathway. The anatomical arrangement of the olfactory nerve and the olfactory bulb in the nasal cavity and forebrain forms the passage between the nasal epithelium and the CNS which make CoV enter the brain [40]. After infecting nasal cells, the virus enters both the olfactory nerve and olfactory bulb to reach the brain and cerebrospinal fluid [42].

4. Hypoxic injury

The virus will multiply in the lung tissue leading to the disruption of the alveoli. This will lead to hypoxia in the CNS. Acid environment causes brain vasodilation, interstitial edema, cerebral blood flow obstruction, and even headache due to ischemia. In fact, COVID-19 patients often suffer from severe hypoxia which can cause nervous system damage [43].

5. Immunity

Systemic inflammatory response syndrome (SIRS) can be caused by viral infection for example in severe pneumonia [44, 45]. SARS and COVID-19 caused high mortality [46]. The ability of CoV in infecting macrophages, microglia, and astrocytes in the CNS is critical. Neurotropic viruses can activate glial cells and induce a pro-inflammatory state. Interleukin (IL)-6, a component of cytokines [47]. In addition, a study has confirmed that a large number of inflammatory factors are increased CoV infection [48].

6. Other causes

The CNS has a lot of parenchymal structure which is a barrier for viruses to pass through this layer. However, once the virus has gained access to the CNS, it is difficult to eliminate it. Lacking the concentration of complex antigens on nerve cells, virus elimination in nerve cells depends solely on the role of cytotoxic T cells [48].

Some mechanisms of nervous system damage due to the COVID-19 infection process certainly play a role in the development of neuropathic pain. This is associated with the fact that neuropathic pain results from disease or injury to the peripheral or central nervous system and lesions can occur at any point. These damaged nerve fibers send the wrong signal to other pain centers which can develop into neuropathic pain [10, 31].

### Pain management in COVID-19 patients

The initial symptoms of COVID-19 infection in general are muscle and body aches, as well as fever, persistent dry cough and other flu-like symptoms. The British Pain Society (BPS) recommends that people with COVID-19 symptoms, who need further advice regarding pain symptoms, should seek help by contacting the National Health Service (NHS) coronavirus service, before they contact their doctor, local pain clinic, or other health professionals [49].

### Immune responses and opioid therapy

Chronic pain plays a complex role on the immune system. Significant immune system changes occur to COVID-19 patients, and studies revealed an altered immune system, with a higher mortality, and were found in the elderly and individuals with comorbid such as hypertension, diabetes, coronary artery disease, and chronic lung disease. Opioids act on the hypothalamus-pituitary-adrenal axis and the autonomic nervous system which involves both innate and acquired immune responses. Greater endocrine abnormalities were found commonly in a patient with higher doses and longer duration of treatment [49].

Morphine and fentanyl have been observed to be the most immunosuppressive among others. Based on the existing data, buprenorphine appears to be the safest for elderly patients who are prone to infection. Moreover, observational studies found higher incidence and severity of infection in patients hospitalized with opioids. Therefore, chronic pain sufferers who use opioids are prone to COVID-19 infection and other secondary infections. In addition, the potential for respiratory depression was higher in hospitalized patients who used the fentanyl patch, because fever increased absorption. High body temperature or fever is often associated with COVID-19 infection, and this can increase the uptake of the transdermal opioid patch and opioid side effects. Patients who are prescribed transdermal opioids and who become increasingly drowsy or somnolent can have their doses reduced, or replaced with short-acting opioid formulations, until the person feels better and the fever subsides. Opioids are also cough suppressants, and they can mask or delay the initial symptoms of COVID-19 infection. The fatigue, nausea, and gastrointestinal symptoms associated with COVID-19 infection can be exacerbated by prescription opioids and other drugs for neuropathic pain [50].

### Anti-inflammatory drugs

Non-steroidal anti-inflammatory drugs (NSAIDs) have been used commonly by many people for controlling pain. Their mechanism of action in analgesic inhibits the synthesis of peripheral prostaglandin through the enzyme cyclo-oxygenase. There are two different forms of the cyclooxygenase enzyme: COX-1 which is expressed in normal cells and COX-2 which is induced in inflammatory cells. The kinin-prostaglandin system associated with angiotensin-converting enzyme (ACE) action as anti-hypertensive agent. Following a report from the British Pain Society on March 14, 2020, there are still doubts about the use of ibuprofen and non-steroidal anti-inflammatory drugs (NSAIDs) to manage fever and pain in people with suspected COVID-19. It has been suggested that NSAIDs may increase complications from simple respiratory infections or delay recovery from infection [49].

Currently, there is no strong evidence to confirm that ibuprofen increases the chance of catching the virus or worsening symptoms. However, its anti-inflammatory and anti-pyretic effects can mask the symptoms and signs of COVID-19 infection. The Medicines and Healthcare products Regulatory Agency (MHRA) and the National Institute for Health and Care Excellence (NICE) are rapidly assessing the evidence. Currently, patients who have been confirmed to have COVID-19 infection or suspected COVID-19 infection should use paracetamol instead of ibuprofen [49]. The World Health Organization (WHO) reported that there are 73 reviewed studies (28 studies in adults, 46 studies in children, and one study in adults and children). The research deals with acute respiratory infections or conditions commonly caused by respiratory viruses, but none of them specifically address COVID-19, SARS, or MERS. WHO concludes that there is currently no evidence of severe side effects, acute healthcare utilization, long-term survival, or quality of life in people with COVID-19, as a result of NSAID use [50]. Another report by Beth Russell claimed that there is currently no conclusive evidence in prohibiting the use of NSAIDs in the treatment of people with COVID-19 [51]. Based on this, if a person has been prescribed ibuprofen or another NSAID and is taking it regularly, it is advisable not to stop taking the drug without seeking advice from a health professional [49].

NSAIDs are often recommended to relieve fever and symptoms in influenza and COVID-19. However, some reports said that the use of NSAIDs is known to have potential effects on the stomach and kidneys in groups considered to be at a higher risk of developing COVID-19. Patients who are at high risk for NSAID therapy complications are the elderly ones (> 65 years), have a history of gastroduodenal ulcers or gastrointestinal perforation or bleeding, or are diagnosed with Barrett’s esophagus in the last 5 years, long-term use of drugs in chronic inflammatory disease (rheumatoid disease, osteoarthritis either who are severe, persistent, or inflammatory), have comorbidities (diabetes mellitus, chronic obstructive pulmonary disease), and concomitant use of anticoagulants in patients who need chronic anti-inflammatory therapy [52–54].

Acute pain management asserted that the risk ratio of using NSAIDs to the incidence of perforations, ulcers, and bleeds (PUBs) is 2.7 higher than patients who do not take NSAIDs. In addition, the short-term use of COXIB was of similar value to that of placebo in the formation of gastric ulcers. The use of COXIB in causing gastrointestinal side effects was reduced by 55%. The use of proton pump inhibitors (PPI) in this regard can significantly reduce the incidence of peptic ulcers associated with NSAID use. The recommended strategy is to use a combination of COXIB and PPI [52–55].

### Corticosteroid and interventions

Corticosteroids are often used in pain injection procedures including trigger point injections, and they can decrease immune function. The effect of corticosteroids on the immune system of COVID-19 patients is unknown, but concerns have been raised about decreased survival benefits and possible side effects, including avascular necrosis, psychosis, diabetes, and delayed viral clearance. Complications associated with COVID-19 and mortality are higher in some groups of people, especially elderly people and those with comorbidities [50].

Chronic pain sufferers can use oral steroids or receive steroid injections for various musculoskeletal conditions. Methylprednisolone is one the steroid which is used for chronic pain. In a large retrospective study, the patient who takes corticosteroid injection into the joint was prone to have influenza. Overactive immune response was shown in the pathophysiology of COVID-19. Steroid usage in people with COVID-19 is suggested for the patient in the state of refractory shock. With these considerations in mind, we argue that any new therapies that could affect the course of the COVID-19 disease should be taken into consideration with doctors who treat the infectious diseases. It should also be recognized that steroids are used in many diseases routinely although there is no evidence to support this practice [50].

### Psychological, physical, and social functions

Chronic pain patients more common to have anxiety, depression, and even suicidal ideation, which can be worsened during this pandemic. Moreover, they also have a stigma, financial stress, and lost their identity which then ended in social isolation that can impact their psychological health, social circumstances, and ongoing chronic pain. These issues can be solved by using a biopsychosocial model of pain management [50].

### Considerations and therapeutic recommendations

The consideration and therapeutic recommendations for chronic pain management during the COVID-19 pandemic can be in many aspects, such as the use of telemedicine; the biopsychosocial management in pain; the procedures in elective pain; the usage of opioid, steroids, and non-steroidal anti-inflammatory drugs; and the procedures in semi-urgent visits [50].

### Telemedicine

Telehealth and telemedicine can give telehealth visits, virtual meetings, and electronic visits, which can decrease the cost and save time for patients. Internet-based platforms including smartphone apps have given an effective method in psychotherapy especially during a public health crisis. These methods also have some limitations such as the information given in Internet-based platforms needs to be adjusted culturally. Health care providers should be aware of licensing requirements in their practice and acknowledge that some rules and regulations that have been canceled during the COVID-19 pandemic will be reversed once the pandemic is resolved [50].

### Biopsychosocial management

Chronic pain patients are advised to consult their overwhelming pain to the psychologists and physical therapists. Multidisciplinary pain self-management programs and strategies for pain self-management can be posted online. Studies founded that cognitive-behavioral therapy give an excellent results for chronic pain. Improving sleep hygiene, practicing mindfulness, doing pacing activities, having a healthy lifestyle, and participating in simple physical exercise and social support can be some of interventions that can be done effectively over the Internet for management of stress in a patient with chronic pain [50].

### Elective pain procedures

In order to decrease the number of hospital visitors during this pandemic, many hospitals have limited or stopped performing the injection procedures. This is due to the need of freeing up capacity, both in terms of time and professional beds of health service, and to better meet the urgent need to treat patients with COVID-19-related symptoms. Therefore, it is likely that there will be an inevitable delay in the non-urgent care of sufferers with persistent pain [50].

### Opioid prescribing

Doctors and other health professionals should evaluate, initiate, and continue opioid prescriptions by using telemedicine. The health professionals should make sure all patients are given the correct opioid to avoid withdrawal. For patients who are prone or have any high-risk comorbids should be educated for naloxone’s prescription. Besides, the risks and effects of a long-term opioid usage need to be educated also to the patients [50].

### Principles of non-steroidal anti-inflammatory drug use

Health professionals should educate the side effects of NSAIDS usage for all patients who are taking non-steroidal anti-inflammatory drugs regularly. Moreover, for patients who are taking non-steroidal anti-inflammatory drugs, they should tell their health care immediately if they have a mild fever or new myalgia because of these drugs [50].

### Principles of steroid use

Steroids play a role in increasing the potential of adrenaline adequacy and damage the immune response. As an example, the use of intra-articular steroid injections is associated with a higher risk of viral infection. This adverse effect could be avoided by evaluating the risks and benefits of steroid injections used and using minimal dose [50].

### Procedures in semi-urgent visits

There is a need for a comprehensive evaluation and a need to help the patient in making decisions. Remote medication can be used to evaluate the patients, determine the emergency, and make appropriate treatment, which will minimize delays and avoid unnecessary visits.^51,^

## Conclusion

Pain complaints that are often found among COVID-19 patients are muscle pain, in contrast to neuropathic pain which is rarely reported. The mechanism of COVID-19 infection in the skeletal, muscular, and nervous system damage is currently believed to be due to the expression and distribution of ACE-2. Some pain management recommendations such as ensuring the care continuity, painkillers, use of telemedicine, and a biopsychosocial management approach have been developed. Nevertheless, it needs to be highlighted that these are not guidelines, so adaptation to the policies of each region is essential in this case.

## Abbreviations

COVID-19: Coronavirus disease 2019
ACE-2: Angiotensin-converting enzyme 2
WHO: World Health Organization
IL-2R: Interleukin 2R
SIRS: Systemic inflammatory response syndrome
BPS: British Pain Society
NHS: National Health Service
NSAIDs: Non-steroidal anti-inflammatory drugs
MHRA: The Medicines and Healthcare Products Regulatory Agency
NICE: The National Institute for Health and Care Excellence
